# Voluntary Age Regression Entering “Headspace” in a Child With Post-traumatic Stress Disorder

**DOI:** 10.7759/cureus.22131

**Published:** 2022-02-11

**Authors:** Alexander J O'Donovan, Hector Cardiel Sam, Jasmin G Lagman

**Affiliations:** 1 Child and Adolescent Psychiatry, Penn State College of Medicine, Hershey, USA; 2 Child and Adolescent Psychiatry, Penn State Health Milton S. Hershey Medical Center, Hershey, USA

**Keywords:** effects of social media, adolescent, coping mechanisms, age regression, posttraumatic stress disorder

## Abstract

In patients with post-traumatic stress disorder (PTSD), the use of coping mechanisms seems to correlate with higher levels of resiliency; however, in the age of the internet, patients may find it easier to discover new unhealthy skills that can hinder their treatment and further progress their symptoms. This report describes the case of a 12-year-old female with PTSD who was admitted for suicidal ideation and who presented with age regression that was voluntary in nature, characterized by reverting to the age of a six-year-old girl while her boyfriend took on a parental role for her. These behaviors were learned through her use of social media. This case demonstrates the use of maladaptive behaviors to cope with their trauma and the need for parental supervision on the use of the internet and social media by the younger population.

## Introduction

The Diagnostic and Statistical Manual of Mental Disorders 5 (DSM-5) states that post-traumatic stress disorder (PTSD) has the essential feature of the "development of characteristic symptoms following the exposure to one or more traumatic events." In some individuals, the symptoms may reflect more fear-based and emotional behavior symptoms, while others may have anhedonic or dysphoric mood states predominate [1]. In people with PTSD, high levels of resilience are associated with a person's use of coping skills. According to Thompson et al., coping can be described as using cognitive strategies to alter one's environment or avoid internal stressors. These coping skills can be active or avoidant, choosing whether to internally modify the perceptions of the trauma or ignore it, respectively [2]. Some researchers argue that addressing and modifying these avoidant behaviors may reduce symptoms of PTSD [3,4]. However, further investigation is needed on the topic. This case report serves as an example of a unique avoidant coping mechanism in response to a childhood trauma.

## Case presentation

A 12-year-old female child with past psychiatric diagnoses of PTSD, anxiety, and depression presented to the inpatient facility with worsening depression, anxiety, and suicidal ideation. She had been having suicidal ideation for the past two months and eventually attempted suicide by overdosing on hydroxyzine due to family and school stressors. She endorsed depressed mood, poor energy, difficulty concentrating, irritability, changes in sleep (both increased and decreased depending on the day), poor appetite, anhedonia, increased guilt, and suicidal thoughts. The patient reported a history of sexual trauma within the past several months that she was uncomfortable disclosing to her treatment team, and Children and Youth Services were investigating the allegations at the time of admission. 

The patient shared with the team that when she was feeling stressed, anxious, or was experiencing flashbacks, she entered "headspace." She defined "headspace" as a mental space where she would become a "little," voluntarily regressing to the mental state of that of a young child around the age of six. She accomplished this by performing activities that someone at the desired age would perform, such as coloring, playing with clay, or cuddling. Her then 14-year-old boyfriend would also participate in these acts, playing the "daddy" figure who consoled and cared for the patient while she was in "headspace" and calling her names like "little princess," "little kitty," and "daddy's little girl." He would also purchase items like pacifiers and baby bottles for her to use while in this state. 

The patient's mother believed that there was a sexual nature to this voluntary age regression and believed that both the patient and her boyfriend were partaking in daddy dom/little girl (DDLG) role-play. According to the mother's research, DDLG is a form of sexual role play in which each member in the relationship takes on one of two roles: the "daddy dom," who acts as a caretaker, and the "little girl," who regresses in age to act of a child-like girl. The patient became defensive when this accusation was made and maintained that it was strictly non-sexual. She believed that it was therapeutic to return to an age before she experienced any of her traumatic events, and she said that she learned about it from her friends who also perform the activity, from social media apps such as Instagram, and from websites she discovered through Google searches on the topic. She was not ashamed of this coping mechanism, portraying her status as a "little" on the artwork she created during group sessions and talking about it openly amongst her peers with excitement and pride.

The patient was previously diagnosed with anxiety and depressive disorders and was seeing a therapist for the past few months. There were no significant medical issues. Family history was significant for a history of anxiety in a second-degree relative. There were no issues with developmental history, and social history was significant for parental separation within the past two years. Her parents do not get along well. The patient's mother's boyfriend moved in with them a few months prior to this admission. She was attending regular classes, but her grades were deteriorating. 

During this hospital stay, she reported classic symptoms of trauma, which included nightmares as well as flashbacks of witnessing domestic violence in the home, with an inability to intervene. She wanted all avoidance with the alleged perpetrator, and she had persistent symptoms of insomnia, irritability, and difficulty concentrating since experiencing her trauma. Once she became more comfortable with the treatment team, she provided additional details on the previously reported sexual trauma. Individual therapy and family meetings focused on the unstable home environment as well as her fear of being in contact again with the alleged perpetrator. As she was already on fluoxetine, this medication was continued, and the dose was increased to address the worsening of depression and anxiety. Individual and family therapy focused on safety planning and addressing her fears, leading to improvement in her anxiety. Extensive psychoeducation was done regarding the danger of excessive reliance on social media and the use of unreliable therapeutic interventions by non-experts or websites and how it can negatively impact her treatment. This was initially faced with resistance. Resolution of her age regressive behaviors was noted after family meetings where she was able to express her fears and concerns to her mother. They expressed understanding that her preferred coping mechanism was non-evidenced based, after which there were clinical improvements. Trauma-focused cognitive-behavioral therapy (TF-CBT) was recommended in the outpatient to address the ongoing symptoms of PTSD, with which she agreed to participate. 

## Discussion

As exhibited by her known traumatic event, nightmares, preferred avoidance of the alleged perpetrator, decreased concentration, and increased irritability, we believe our patient fits the ICD-10 criteria for PTSD [5]. Current treatments for PTSD consist of pharmacotherapy, psychotherapy, or a combination of both. For pharmacotherapy, selective serotonin reuptake inhibitors (SSRIs) are generally regarded as the first-line therapy, specifically for fluoxetine or paroxetine. The serotonin/norepinephrine reuptake inhibitor venlafaxine is also seen as a common recommendation for first-line pharmacotherapy [6]. A systematic review conducted by Martin et al. in 2021 found that one-third of guidelines recommended psychotherapy over pharmacotherapy, and all chose cognitive-behavioral therapy (CBT) as first-line [6]. Our patient was maintained on fluoxetine while inpatient and recommended for trauma-focused CBT outpatient as part of her therapy per these guidelines.

There is limited literature on DDLG and voluntary age regression. The available information on the web comes mostly from personal accounts of people or groups engaging in these behaviors. However, the idea of regression as a response to a traumatic experience is certainly not new. Studies have described adolescents developing late-onset secondary enuresis following a traumatic experience, such as a car accident [7,8]. Other common regressive behaviors seen by hospitalized psychiatric patients include crying, engaging in baby talk, sucking on objects or body parts, assuming the fetal position, and needing a comforting object, like a stuffed animal, among others [9]. These regressive behaviors seen in hospital patients are usually involuntary and are a result of various psychiatric disorders such as major depressive disorder, catatonia, psychotic disorders, delirium, borderline personality disorder, or substance abuse disorders. While studies addressing the prevalence of regression in hospitalized patients are lacking, the number of patients referred to as "agitated" while inpatient can also help infer that number of regressing patients may be misclassified [9]. 

The difference between our patient's presentation of regression and the regression as described in the existing literature is that the former is voluntary. Where regression may be a clinical feature of many medical, psychiatric, or neurologic disorders, age regression, when done voluntarily, may hinder treatment. Negative coping skills learned from the internet or friends, with no therapeutic studies done in the past, can be detrimental to patients. In the case of this patient, she found herself in an abusive situation with her partner because of this dominant role he had over her decisions. She avoided talking about different stressors in her life with people other than her partner, including her mother, with whom the patient still claimed she felt close. Instead, she repressed her traumas and stressors and underwent voluntary age regression by entering a "headspace." This act of pretending to be a different age and ignoring the trauma and stressors in her life hindered her ability to develop positive coping mechanisms and benefit from her initial management of her PTSD. 

PTSD is becoming a growing problem for children, adolescents, and college-aged individuals [10,11]. Kilpatrick et al. found in 2003 that the six-month prevalence was estimated to be 6.3% in female adolescents and 3.7% in male adolescents [12]. As numbers of children and adolescents with PTSD increase over time, new variations of coping mechanisms may manifest, both positive and negative. A study found that all avoidant coping strategies were negatively correlated with the concept of resilience and positively correlated with further progression of PTSD symptoms [2]. As seen in this case, subordination and submissiveness are also associated with increased anxiety and depression [13].

As clinicians, it is important to acknowledge some of the coping mechanisms patients will adopt to manage their trauma. As the growing influence of the internet and subgroups that can be found on it expands, people will find ways to use it to cope with their stressors. Studies have found that those with problematic internet use are more likely to have maladaptive coping strategies and use immature, neurotic, and autistic fantasy defenses [14]. Understanding some of the ways the internet can influence patients to try new and possibly negative coping mechanisms is important for clinicians to be aware of to better understand their patients and provide appropriate and timely intervention. 

## Conclusions

PTSD is becoming more frequently diagnosed in children and adolescents in recent years. Coincidentally increasing in recent years has been society's use of the internet. As this happens, patients living with PTSD may gain access to coping mechanisms that could be unhealthy in nature and lead to further harm. Voluntary age regression and submissiveness are techniques that can easily be discovered through the internet and social media and are two of those starting to be used by adolescents as a method of self-management for their stressors. Since no therapeutic studies have been done on these techniques, they can potentially worsen PTSD symptoms and negatively impact a patient's treatment and recovery. Healthcare professionals, parents, and other personnel working with children and adolescents may benefit from being aware of these negative techniques used by our younger population so that they may help guide them to seek more evidence-based practices.
